# Characterization of the genetic and regulatory networks associated with sugar and acid metabolism in apples *via* an integrated strategy

**DOI:** 10.3389/fpls.2022.1066592

**Published:** 2022-11-17

**Authors:** Fei Shen, Chenyang Hu, Xin Huang, Ruigang Wu, Shuzhen Luo, Chengnan Xu, Hong Zhang, Xuan Wang, Jirong Zhao

**Affiliations:** ^1^ Institute of Biotechnology, Beijing Academy of Agriculture and Forestry Sciences, Beijing, China; ^2^ College of Life Science, Shanxi Key Lab of Chinese Jujube, Yan’an University, Yan’an, Shanxi, China; ^3^ College of Landscape and Ecological Engineering, Hebei University of Engineering, Handan, Hebei, China; ^4^ Hebei Normal University of Science & Technology, Qinhuangdao, Hebei, China

**Keywords:** apple, sugar, acid, RNA-seq, QTL, integrated strategy, genome sequencing

## Abstract

Although sugars and acids have a substantial influence on the taste of apple fruits, the genetic and regulatory networks underlying their metabolism in fruit remain insufficiently determined. To fully decipher the genetic basis of the accumulation of sugars and acids in apple fruits, we adopted an integrated strategy that included time-course RNA-seq, QTL mapping, and whole-genome sequencing to examine two typical cultivars (‘HanFu’ and ‘Huahong’) characterized by distinctive flavors. Whole-genome sequencing revealed substantial genetic variation between the two cultivars, thereby providing an indication of the genetic basis of the distinct phenotypes. Constructed co-expression networks yielded information regarding the intra-relationships among the accumulation of different types of metabolites, and also revealed key regulatory nodes associated with the accumulation of sugars and acids, including the genes *MdEF2*, *MdPILS5*, and *MdGUN8*. Additionally, on the basis of QTL mapping using a high-density genetic map, we identified a series of QTLs and functional genes underlying vital traits, including sugar and acid contents. Collectively, our methodology and observations will provide an important reference for further studies focusing on the flavor of apples.

## Introduction

Apples (*Malus* × *Domestica* Borkh.) are among the most commercially important fruit crops cultivated worldwide with a considerable global production (FAOSTAT, http://faostat.fao.org). With increasing market demand, consumers are pursuing higher quality apple fruits with good flavors, reflecting desirable sugar and acid contents.

Among the quality-related traits of apple fruit, perhaps the most important are appearance, flavor (Cappellin et al., 2015; Guan et al., 2015; Xu et al., 2022), and texture (Sara et al., 2013; Bink et al., 2014), which have been established to be quantitative traits controlled by multiple genes. With respect to flavor, the contents of sugars and acids in apple fruits have been particularly well studied. For example, the *MdbHLH3* gene has been demonstrated to regulate the expression of *MdPFPβ* to promote sugar accumulation (Yu et al., 2022) and regulate the expression of *MdcyMDH* to enhance acid content (Yu et al., 2021). Similarly, the expression of *MdTSTa* and *MdMa11* has been found to promote the accumulation of sugars and acids in fruits (Ma et al., 2021). Conversely, the expression of *MdSUT4.1* has typically been observed to be negatively correlated with the accumulation of fructose (Peng et al., 2020). Furthermore, it has been established that *MdERDL6-1* can influence the accumulation of glucose in vesicles by regulating the expression of two types of transporter protein, MdTST1 and MdTST2 (Zhu et al., 2021), whereas transient overexpression of *MdVGT1* and *MdpGlcT2.1* promoted significant increases in glucose concentration (Zhu et al., 2022), and *MdWRKY126* has been observed to influence the accumulation of malic acid in apple fruits by regulating the expression of *MdMDH5* (Zhang et al., 2022).

RNA-Seq is an effective technique that can be used to examine gene function and investigate vital trait-related biological pathways, and in recent years, RNA-seq methodology has been widely applied to characterize gene function in different crop species such as pepper (Park et al., 2019), oilseed rape (Jian et al., 2019; Song et al., 2021), tomato (Wen et al., 2019), and maize (Xu et al., 2022). By deciphering the co-expression modules associated with particular traits, we can potentially gain an in-depth understanding of the underlying gene regulatory networks. For example, an examination of the effect of low temperature on anthocyanin accumulation, revealed the genes *MdMYB22*, *MdMYB12* and *MdMYB114* to be specifically expressed within co-expression modules highly associated with anthocyanin accumulation (Song et al., 2019). Similarly, the *Ma1* gene identified in the “MEturquoise” module highly associated with acidity provided insights for the study of fruit acidity (Bai et al., 2015). In a parallel bud mutation study, several candidate genes were identified in modules associated with sugar and acid specificity, including *MdDSP4*, *MdINVE*, and *MdSTP7*, which play important roles during fruit development (Zhao et al., 2019), and the discovery of these genes has made an important contribution to the current focus of studies examining the accumulation of sugars and acids in bud mutation.

Quantitative trait locus (QTL) mapping has been widely employed to determine the genetic basis of important quantitative traits in apples. For example, three major QTLs associated with volatile organic compounds were detected in the progeny population derived from a ‘Fiesta’ × ‘Discovery’ cross (Costa et al., 2013), and the regulatory gene *MdSDH2*, which controls the fructose content in fruits, was detected using an F_1_ population obtained from a cross between ‘Honeycrisp’ and ‘Qinguan’ (Wang et al., 2022). Furthermore, four QTLs distributed on chromosomes 8 and 16 were found to be associated with fruit acidity using a combined MapQTL and BSA-seq approach, and it has been demonstrated experimentally that *MdSAUR37*, *MdPP2CH*, and *MdALMTII* influence the malic acid content in fruits (Jia et al., 2018). Moreover, QTL analysis of different polyphenolic compounds among the progeny of a ‘Royal Gala; × ‘Braeburn’ cross identified *LAR1* and *HCT/HQT* as important enzymes affecting the concentrations of polyphenolic compounds in apple fruit varieties (David et al., 2012; Sun et al., 2015). However, despite these important discoveries, completely deciphering the associated genetic and regulatory networks remains a challenge.

Motivated by the findings of recently reported studies, we have adopted multi-level strategies, including genome sequencing, RNA-seq, and QTL mapping, to further decipher the genetic basis and regulatory networks associated with sugar and acid metabolism during the development of apple fruit. In this study, we examined the changes in sugar and acid contents in fruits of the cultivars ‘Hanfu’ and ‘Huahong’ at different time points during fruit development, analyzed the results based on whole-genome sequencing to assess the amount of variation between these two cultivars, and applied RNA-seq technology to elucidate the regulatory networks associated with sugar and acid metabolism. To identify candidate genes associated with sugar or acid metabolism, we compared the genes identified within the detected QTL intervals with those shown to be differentially expressed based on RNA-seq analysis The findings of this study provide new insights into the genetics and regulation of fruit sugars and acids from different perspectives, and will serve as a valuable basis for further research on sugars and acids in apple fruits.

## Materials and methods

### Plant materials and sampling

The two apple cultivars ‘Hanfu’ and ‘Huahong’ and 210 offspring used in this study were planted in the Liaoning Institute of Pomology, Xiongyue, Yingkou, Liaoning, China (40°17ʹN, 122°15ʹE). The fruits were harvested at 30, 90, and 150 days after blooming (DAB) with three biological replicates. The classification of apple fruit developmental stages was based on previous research, with the three selected time points corresponding to the juvenile, expansion, and maturity stages, respectively (Janssen et al., 2008). Each harvested apple was peeled, cut into pieces, and then frozen in liquid nitrogen and stored at -80°C.

### Determination of sugar and acid content in fruit

For each fruit, we measured the sugar and acid contents. Samples (10 g) were ground to a fine powder using a SPEX 6870 lyophilizer (SPEX, Metuchen, USA) under liquid nitrogen and extracted with ultrapure water. The extract thus obtained was centrifuged at 16000 × *g* for 10 min and the resulting supernatant was passed through an OnGuard II Ag column (Dionex Corporation, Sunnyvale, CA, USA). The extracts were filtered using a Sep-Pak filter containing a 0.22-μm aqueous membrane. The sugar and acid contents of apple fruits were determined using a DIONEX ICS-5000 high-performance liquid chromatography system (Dionex Corporation, Sunnyvale, CA, USA). In addition, for each fruit, we determined the fruit weight, fruit diameter, fruit length, fruit shape index, flesh firmness, soluble solids, and flesh browning of freshly harvested samples. Data were analyzed using Graphpad Prism software (V8.2.1) and an analysis of variance (ANOVA) was used to test for differences between groups.

### Resequencing of ‘Hanfu’ and ‘Huahong’

Genomic DNA was extracted from fruit samples using a Genomic DNA Isolation Kit (TianGen, Beijing, China). Illumina sequencing libraries were constructed using NEBNext DNA Library Prep Mix (NEB), and paired-end sequencing was performed using an Illumina HiSeq X ten platform (Illumina, San Diego, CA, USA). Clean reads were mapped to the doubled haploid (DH) apple genome using the Burrows–Wheeler Aligner (version 0.7.17) (Janssen et al., 2008; Daccord et al., 2017), SNP and InDel calls were processed using SAMtools software (Li et al., 2009a; Li, 2011), and structural variant calling was performed using Delly software (version 0.8.1) (Rausch et al., 2012).

### RNA-Seq library construction, sequencing, and data processing

Total RNA was extracted using a modified CTAB method. An NEBNext Poly(A) mRNA Magnetic Isolation Module and NEBNext Ultra Directional RNA Library Prep Kit for Illumina (New England Biolabs, Massachusetts, USA) were used to isolate mRNA for RNA-seq library preparation. cDNA libraries were sequenced using the Illumina HiSeq X Ten platform (Illumina). HISAT2 was used to compare quality-controlled data based on the reference genome (Kim et al., 2015), and StingTie was used for transcript assembly and quantification (Pertea et al., 2015). Raw data were extracted from each sample and differentially expressed genes (DEGs) were detected using the default parameters of DESeq2 (Love et al., 2014) software, with genes having a false discovery rate (FDR) of less than 0.01 being identified as differentially expressed.

### Identification of co-expression modules

We conducted weighted gene co-expression network analysis (WGCNA) of the expression data using the R package WGCNA (Zhang and Horvath, 2005; Langfelder and Horvath, 2008). The threshold strength of the correlation matrix was selected according to a pickSoftThreshold function of 8. The resulting adjacency matrix was converted to a topological overlap (TO) matrix using the TOM similarity algorithm, and the genes were hierarchically clustered based on TOM similarity. The hierarchical clustering tree was partitioned using a dynamic hybrid tree pruning algorithm and the branches obtained after tree pruning were defined as modules. We summarized the expression of each module as a first principal component (referred to as the module feature gene). Modules that were highly correlated (a coefficient greater than 0.75) were merged.

### QTL mapping

The genetic map used for QTL mapping was constructed using Specific-Locus Amplified Fragment Sequencing (SLAF-seq) technology (Sun et al., 2013) and HighMap software (Liu et al., 2014) developed by the Beijing BMK Biotechnology Company for high-density molecular marker construction (including resequencing of the two parents and SLAF simplified genome sequencing of 210 offspring) for the genetic segregation population of apple cultivars (unpublished). The map, which included a total of 7043 markers, had a total length of 2804.01 cM (unpublished). The phenotypic data, genotypic data, and number of individual plants were imported into MapQTL 6.0 mapping software, and the QTLs associated with sugar and acid contents were analyzed based on a mixed model and interval mapping, with the 95% confidence interval of LOD values being calculated using the Permutation test of MapQTL 6.0 software.

## Results

### Phenotype characteristics of ‘Hanfu’ and ‘Huahong’ during fruit development

**Figure 1:**
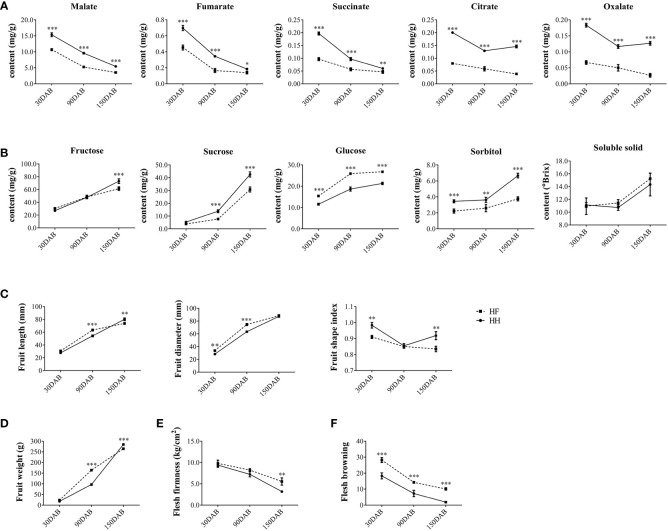
Fruit phenotype at different stages of ‘HanFu’ and ‘Huahong’ fruit development. **(A)** Contents organic acid in fruit of the two cultivars at three stage of development [30, 90, and 150 days after flowering (DAB)]. **(B)** Contents of sugars, sorbitol, and soluble solids in fruits of the two cultivars at the three stages of development. **(C)** Physical measurements of fruit length, diameter, and fruit shape index of the two cultivars at the three developmental stages. **(D–F)** Phenotypes of fruit weight, flesh firmness, and flesh browning showing variation during development. The error bars represent the standard deviation (SD) among the replicates (n = 5). *, **, and *** indicate significant difference between the two cultivars at *P* < 0.05, 0.01, and 0.001, respectively, as determined using Student’s *t*-test.

Fruits of the two important apple cultivars ‘Hanfu’ (HF, ‘Toko’ × ‘Fuji’) and ‘Huahong’ (HH, ‘Golden Delicious’ × ‘Megumi’) were collected for fruit quality assessment at 30, 90, and 150DAB, which correspond to the juvenile, expansion, and maturity stages of fruit development, respectively. We found that concentrations of malate, fumarate, citrate, succinate, and oxalate declined during the course of development. Moreover, at each of the three stages assessed, the contents of all five acids were significantly higher in HH than in HF (**Figure 1A**). In contrast, we detected increases in the concentrations of fructose, sucrose, glucose, sorbitol, and soluble solids from 30 DAB to 150 DAB. Among these sugars, the concentrations of fructose in HH fruit at 150 DAB and sucrose at 90 DAB and 150 DAB were significantly higher than those in HF, whereas at all stages, we detected more sorbitol and less glucose in HH. However, there were no significant differences between the two varieties with respect soluble solids contents (**Figure 1B**). In addition, HH fruit had a higher fruit shape index (fruit length/fruit diameter) and fruit weight than HF at 150DAB (**Figures 1C, D**), and at maturity, HF fruit was found to be firmer than that of HH, whereas the latter was more resistant to browning at all stages (**Figures 1E, F**). Collectively, these findings indicate the distinct flavors of these two cultivars and accordingly suggested differences in the genetic basis of these distinct favors.

### Whole-genome resequencing identified mutations between ‘Hanfu’ and ‘Huahong’

**Figure 2:**
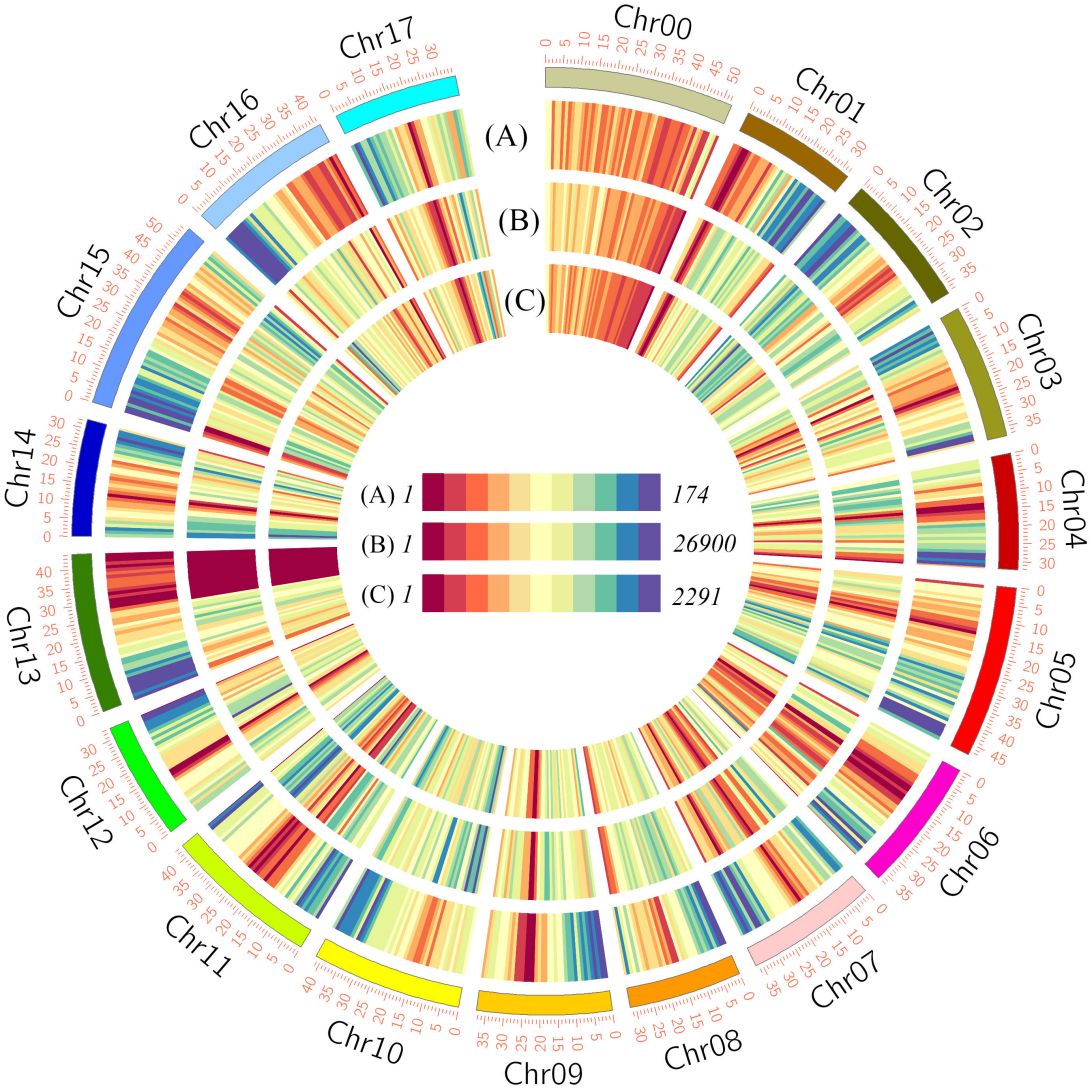
Circular overview of the variations between ‘HanFu’ and ‘Huahong’. The outer circle **(A)** shows the numbers of genes within a 1-Mbp window; the intermediate circle **(B)** shows the number of SNVs between HanFu’ and ‘Huahong’ within a 1-Mbp window; and the inner circle **(C)** shows the number of SVs between ‘HanFu’ and ‘Huahong’ within a 1-Mbp window.

To examine the underlying differences between the two cultivars at the genomic level, we performed whole-genome resequencing analysis, obtaining 153,786,736 and 158,603,659 reads for HF and HH, respectively, with corresponding coverages of 96.20% and 96.64% (**Table S1**). The sequencing reads provided an average 30× coverage of the apple DH genome, with approximately 31.23 million reads assigned to the apple DH genome and approximately 30.12 million reads were uniquely mapped reads (**Table S1**). In total, 9,220,533 SNPs and 737,698 InDels distributed across 17 chromosomes were identified in variant detection analysis of HF versus HH (**Figures 2A, B** and **Table S2**). There were no correlations between chromosome length and the number of SNPs and InDels. For example, although the numbers of both SNPs and InDels were higher on the longest chromosome, Chr15, than on other chromosomes, the lowest numbers of SNPs and InDels were detected on Chr13 and Chr6, respectively (**Table S2**). In addition, the presence of large segmental structure variants (SVs) was detected, which, like SNPs and Indels, were unevenly distributed across the 17 chromosomes (**Figures 2A, C**).

### Transcriptome profiles of ‘Hanfu’ and ‘Huahong’ flesh during fruit development

Using the three replicate fruit samples collected at the three assessed developmental stages (30, 90, and 150 DAB), we performed RNA-Seq analysis to determine differences in gene expression in the flesh of the two cultivars during fruit development. RNA-Seq generated 143.8 gigabytes (Gb) of clean data (Q30 > 93.14%) with 7.00–9.37 Gb obtained from the 18 complementary libraries. After removing low-quality reads, a total of 46,783,954–62,640,562 reads per library were obtained, of which 73.93%–80.89% could be uniquely assigned to the apple DH genome (**Table S3**). All genes were quantified and mapped based on fragments per kilobase transcript per million (FPKM), among which, the expression of 40,134 genes was detected across the three fruit developmental stages (**Table S4**). High Pearson correlation coefficients (>0.95) indicated high quality control among the biological replicates (**Figure S1**). Overall, approximately 24.96%–28.79% of the genes were characterized by low expression, 38.11%–41.19% showed moderate expression, and 25.55%–27.11% and 6.74%–7.07% were identified as highly and very highly expressed, respectively (**Figure S2** and **Table S4**).

### Genes differentially expressed between ‘Hanfu’ and ‘Huahong’ at different stages of fruit development

**Figure 3:**
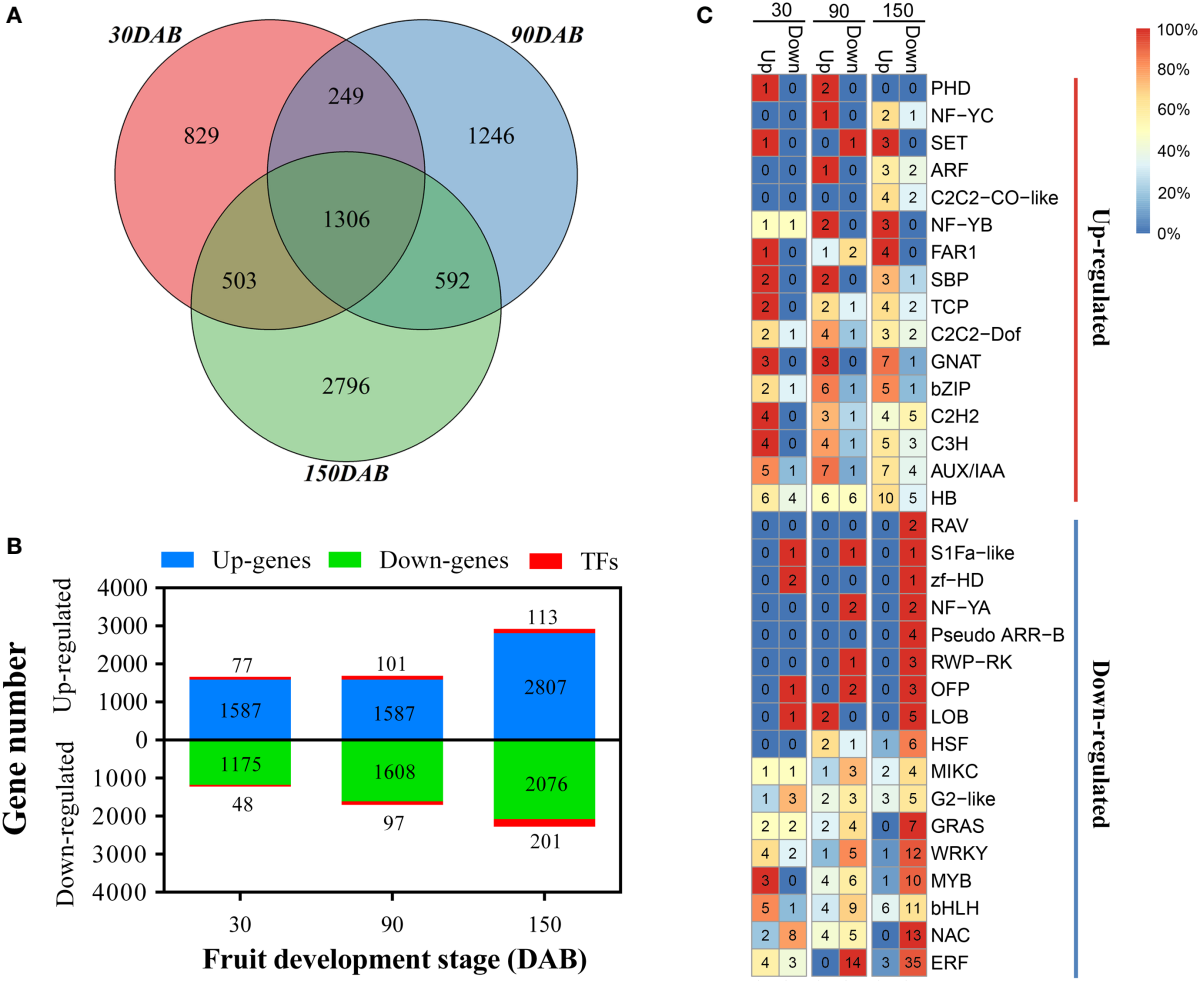
Differentially expressed genes between ‘HanFu’ and ‘Huahong’ at three stages of fruit development. **(A)** A Venn diagram showing the number of differentially expressed genes (DEGs) at the three stages of fruit development. **(B)** A histogram showing the number of up-regulated and down-regulated DEGs and transcription factors (TFs). **(C)** A heatmap showing the number and percentage (color) of up- and down-regulated TFs belonging to different families.

Genes with a greater than 2-fold change and an FDR of less than 0.01 were identified as being differentially expressed using the DESeq R package. In total, we identified 7521 genes showing significantly different expression between HH and HF at the three developmental stages (**Table S5**), with the numbers of DEGs at stages 30, 90, and 150 DAB being 2887, 3393, and 5197, respectively (**Figure 3A** and **Table S5**). Among those genes showing differential expression at 30DAB, 1664, including 77 transcription factor-encoding genes (TFs), showed significantly higher expression, and 1223, including 48 TFs, showed significantly lower expression in HH than in HF (**Figure 3B** and **Table S5**). Comparatively, at 90 and 150 DAB, the numbers of up- and down-regulated genes were 1688 vs. 1705 and 2920 vs, 2277, respectively, including 101 vs. 97 and 113 vs. 201 TFs (**Figure 3B** and **Table S5**). Among the Aux/IAA, GNAT, and bZIP TF families, the numbers of up-regulated members were significantly higher than those of the down-regulated TF members, whereas contrastingly, in the HSF, GRAS, WRKY, bHLH, and zf- HD TF families, the numbers of down-regulated members were significantly higher than those of the up-regulated members (**Figure 3C** and **Table S5**).

**Figure 4:**
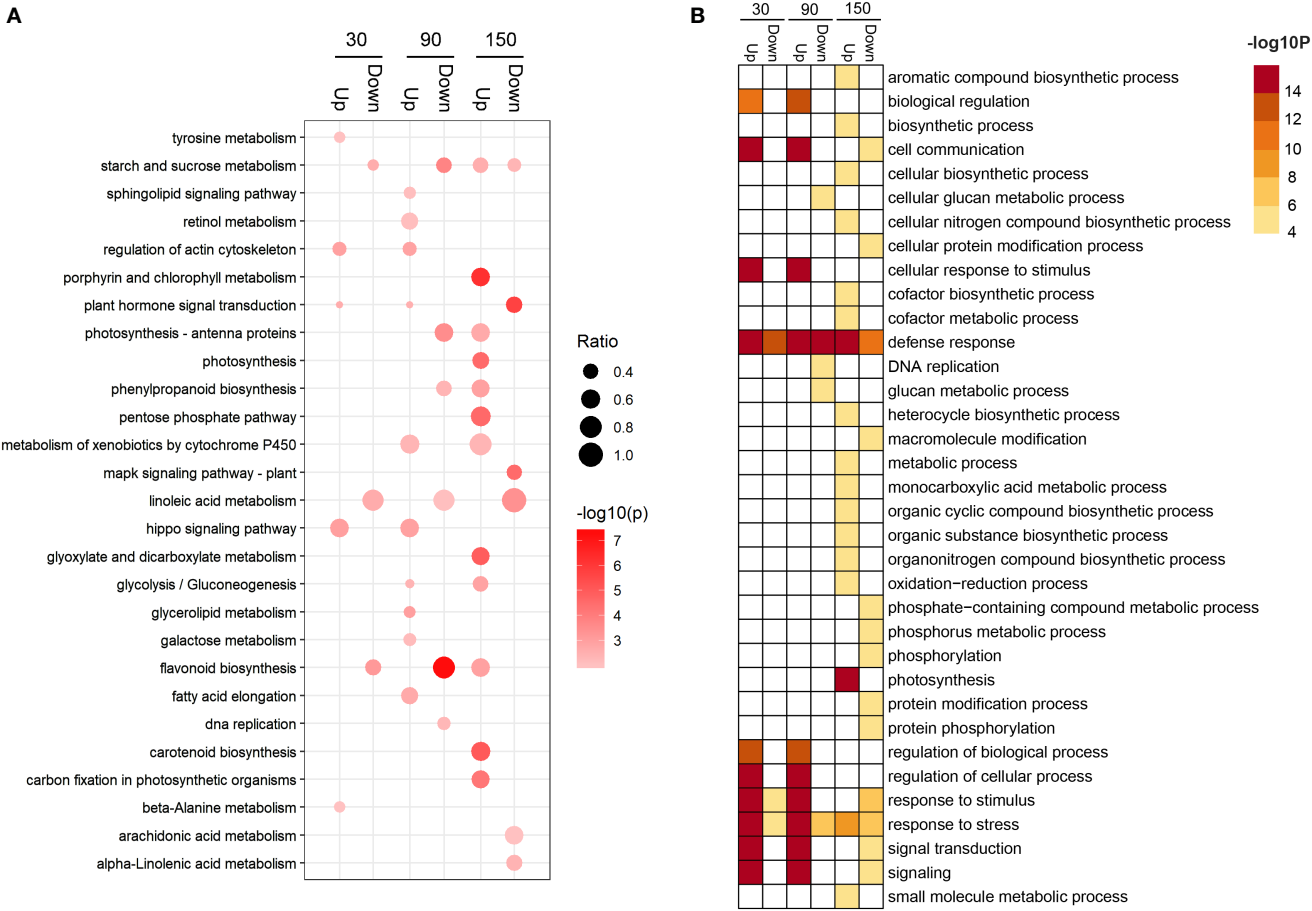
Enrichment analysis of differentially expressed genes between ‘HanFu’ and ‘Huahong’ at three stages of fruit development. **(A)** A bubble diagram showing the result of KEGG pathway analysis of the up- and down-regulated differentially expressed genes (DEGs). The size of the bubbles indicates the ratio of the number of DEGs in the pathway to that of the total number genes identified. **(B)** A heatmap showing the result of GO enrichment analysis of the up- and down-regulated DEGs.

Kyoto Encyclopedia of Genes and Genomes (KEGG) enrichment analysis revealed that differentially expressed genes (DEGs) were significantly distributed among 31 metabolic pathways (P < 0.05) (**Table S6** and **Figure S3**). Among the enriched metabolic pathways, three, namely, starch and sucrose metabolism, plant hormone signal transduction, and flavonoid biosynthesis, reached a highly significant level of enrichment (P < 0.001) (**Table S6** and **Figure S3**). In addition, the starch and sucrose metabolism pathway were enriched in the 30down, 90down, 150up, and 150down gene sets (**Figure 4A** and **Table S7**); the flavonoid biosynthesis pathway was enriched in the 30down, 90down, and 150up gene sets (**Figure 4A** and **Table S7**); and the DNA replication pathway was enriched in the 90down gene set (**Figure 4A** and **Table S7**). Gene Ontology (GO) enrichment analysis of the DEGs identified 49 terms (**Table S8** and **Figure S4**). At 30 and 90 DAB, genes associated with biological regulation and cell communication categories were up-regulated, whereas secondary metabolism and stress categories were enriched at 150 DAB (**Figure 4B** and **Table S9**).

### Identification of co-expression modules among DEGs

**Figure 5:**
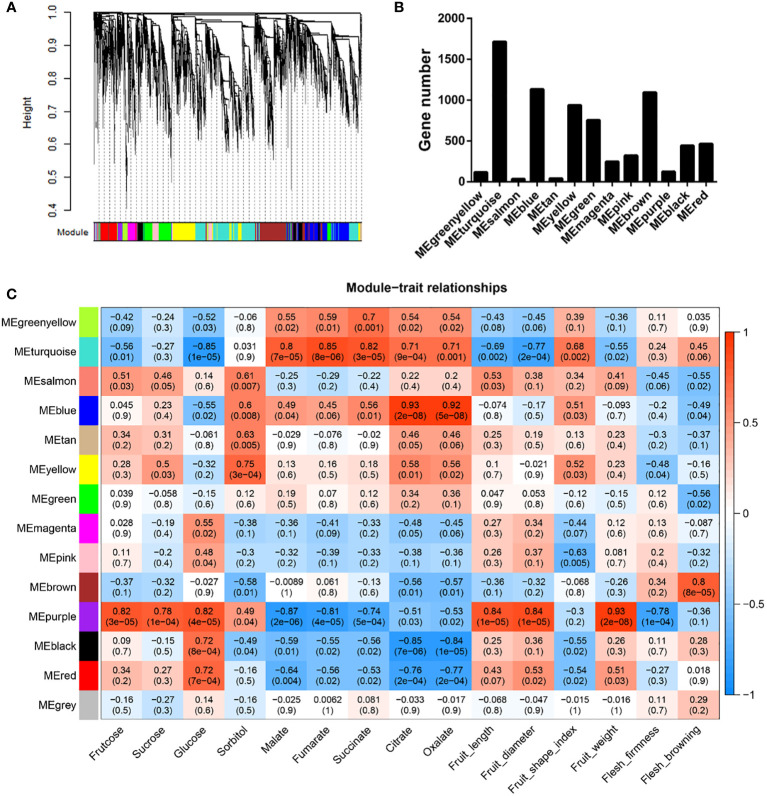
Weighted gene co-expression network analysis of differentially expressed genes between ‘HanFu’ and ‘Huahong’ over three development stages. **(A)** A clustering dendrogram of the differentially expressed genes (DEGs), with dissimilarity based on topological overlap, together with assigned module colors. **(B)** The numbers of genes harbored in each module. **(C)** Module-trait associations. Each row corresponds to a module eigengene, and each column to a trait. Each cell contains the corresponding correlation and P values. The table is color coded by correlation as indicated in the color legend.

To investigate the gene regulatory networks comprising DEGs, we performed weighted gene co-expression network analysis (WGCNA), which accordingly identified 13 modules among the DEGs, with gene numbers ranging from 36 to 1716 (**Figures 5A**, **B**). Module-trait relationship analysis revealed that seven of these modules were significantly associated with at least one property in the 18 samples (|r| ≥ 0.7, P < 0.01) (**Figure 5C** and **Table S10**). The module “MEpurple” was identified as being correlated with most (10) traits (i.e., fructose, sucrose, glucose, malate, fumarate, and succinate contents; fruit length, diameter, and weight; and pulp firmness). The module “MEturquoise” was found to be highly correlated with all assessed organic acids, and the modules “MEyellow” and “MEbrown” were clearly associated with sorbitol content and flesh browning, respectively (**Figure 5C** and **Table S10**). We also identified the following module-trait relationships: “MEblue” - citrate and oxalate; “MEblack” - glucose, citrate, and oxalate; and “MEred” - glucose, citrate, and oxalate (**Figure 5C** and **Table S10**).

### Deciphering key co-expression modules

**Figure 6:**
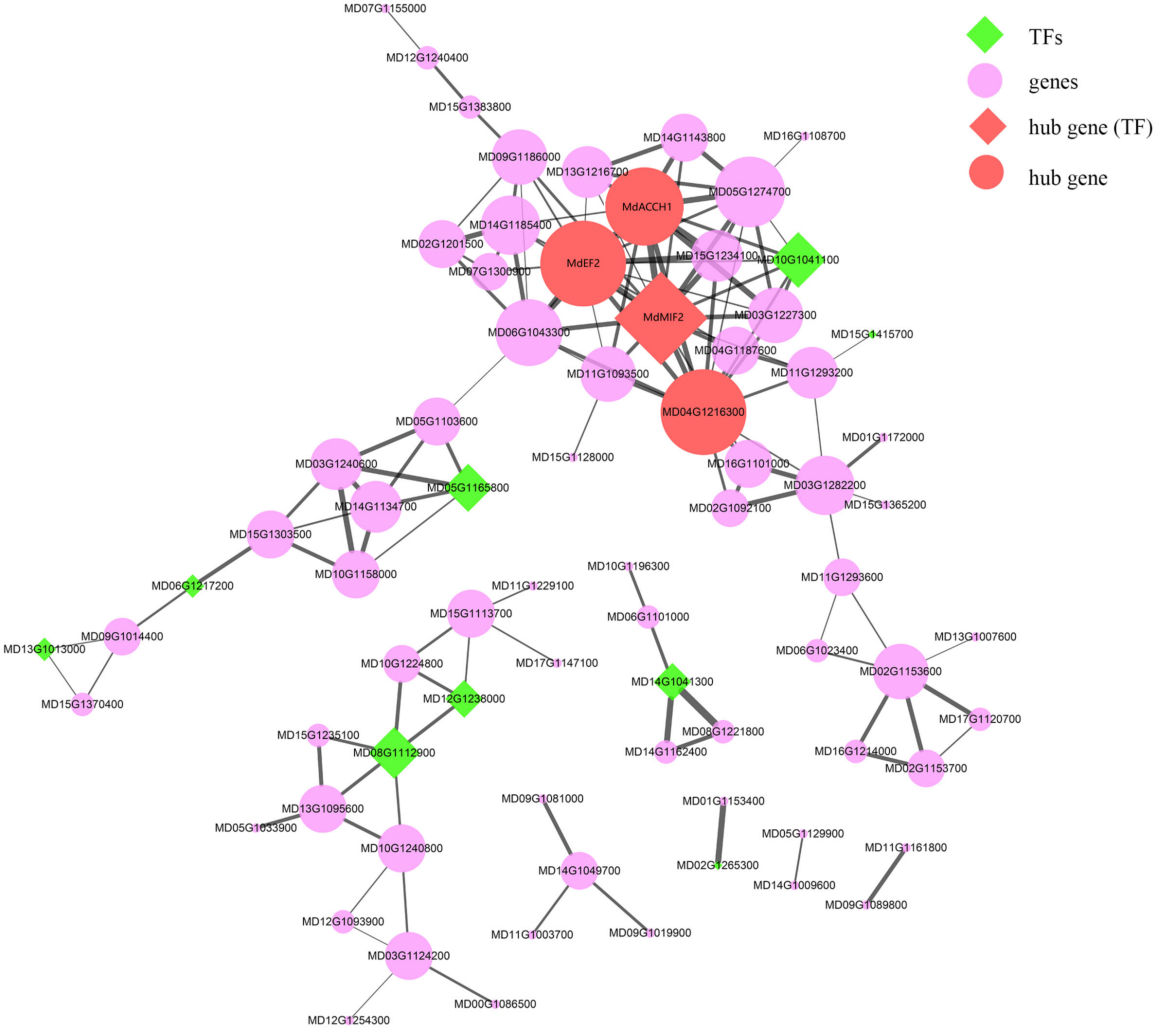
The co-expression network of the “MEpurple” module. The green diamonds indicate transcription factors (TFs) and the red diamonds indicate the hub genes, which are also TFs. The purple and red filled circles denote the genes and hub genes in this module, respectively. The width of edges corresponds to the weight value between different genes, with a larger width being indicative of a larger weight.

As mentioned in the previous section, the “MEpurple” module was found to be significantly correlated with most of the assessed fruit traits (**Table S10**). Within this module, there were 124 genes identified as being differentially expressed between HF and HH, among which, four genes (*MdACCH1*, *MdEF2*, *MdMIF2*, and MD04G1216300) were identified as hub genes (**Table S11** and **Figure 6**). The expression of *MdACCH1*, *MdEF2*, and *MdMIF2* gradually increased during the course of development, reaching peak levels at 150DAB. Contrastingly, MD04G1216300 showed an opposite trend, with the lowest levels of expression being detected at 150 DAB (average FPKM = 29.86). Notably, the *MdEF2* gene, which encodes a ZF-HD transcription factor, showed the greatest difference between the development of HF and HH. These findings thus provide evidence to indicate that *MdEF2* plays an important role in regulating multiple traits in apple fruits.

Module-trait significance analysis revealed that the “MEblack” module, which contained 444 genes differentially expressed between HF and HH during the three developmental periods, was significantly correlated with glucose, citrate, and oxalate contents (**Table S10**). Analysis of corresponding co-expression network revealed three genes (*MdNU160*, *MdRHD32*, and *MdRHD32*) showing a high correlation with other genes in the network, thereby identifying these as the hub genes of the module (**Table S11** and **Figure S5**). Analysis of the expression of these hub genes revealed a gradual increase throughout the development of HF and HH, with *MdRHD32* showing the pronounced change during 30–150 DAB. Combined with our finding that *MdRHD32* was the most strongly associated in the co-expression network, we predicted that *MdRHD32* play an important regulatory role in sugar and acid accumulation during apple fruit development.

The “MEblue” module, comprising 1,134 DEGs, was assessed as showing a high correlation with citrate and oxalate (**Table S10**). Co-expression network analysis identified two hub genes, *MdFTSHC* and MD04G1091300 based on the degree of association with other genes (**Table S11** and **Figure S6**), the expression levels of both of which were up-regulation during all three developmental stages. On the basis of the combined findings of the co-expression network and gene expression analyses, we hypothesized that these two hub genes may play important roles in the accumulation of citrate and oxalate.

The “MEbrown” module, containing 1,096 DEGs, was found to show a significant correlation with fruit flesh browning (**Table S10**). Five genes showing the highest association in the co-expression network, namely, *MdPILS5*, MD14G1011900, *MdGUN8*, *MdPMEI3*, and *MdANRPN*, were identified as hub genes in this module (**Table S11** and **Figure S7**). Analysis of gene expression at the different stages of development revealed that there were different reductions in gene expression during the 30–90 DAB stage, with the changes in *MdANRPN* expression being the most evident. Given the observed changes in *MdANRPN* expression at all developmental stages, we speculate that *MdANRPN* plays an important regulatory role in apple fruit browning.

Similar to the “MEblack” module, the “MEred” module containing 446 DEGs also showed significant correlations for glucose, citrate, and oxalate (**Table S10**). The constructed co-expression network revealed five genes, MD07G1239500, *MdPUB35*, *MdXTH33*, *MdMES17*, and *MdZIP1*, to be strongly associated with other genes, and these were accordingly identified as hub genes of the “MEred” module (**Table S11** and **Figure S8**). Among these genes, the expression of *MdMES17* in HF was found to be significantly higher than that of other genes at the same stage.

The “MEturquoise” module, which contained the highest number of DEGs (1,716 DEGs) was significantly associated with malate, fumarate, succinate, citrate, oxalate, glucose, and fruit diameter (**Table S10**). Co-expression network analysis identified the five genes *MdPSAF*, *MdU603*, *MdRR17*, MD15G1264900, and MD12G1264100 as potential hub genes (**Table S11** and **Figure S9**), among which, the expression of *MdPSAF* was observed to be significantly higher than that of the others. On the basis of differences in the variation of *MdPSAF* expression during fruit development and the strong correlation shown in the co-expression network, we hypothesized that *MdPSAF* has a regulatory effect on the accumulation of glucose and multiple acids in apple fruits.

The “MEyellow” module containing 939 DEGs was found to be specifically associated with fruit sorbitol content (**Table S10**). Co-expression network analysis revealed *MdAB1K8* and *MdRK1* to be the most highly associated genes within the co-expression network, and these were duly identified as module hub genes (**Table S11** and **Figure S10**). Comparative analysis of the gene expression of *MdAB1K8* and *MdRK1* in HF and HH at each of the assessed developmental stages indicated that *MdRK1* plays an important role in the accumulation of sorbitol in fruits.

### Sugar and acid metabolism during apple fruit development

To gain insights into the molecular mechanisms underlying changes in the sugar and acid contents of apple fruit, we analyzed the DEGs identified as being associated with sugar and acid metabolism.

**Figure 7:**
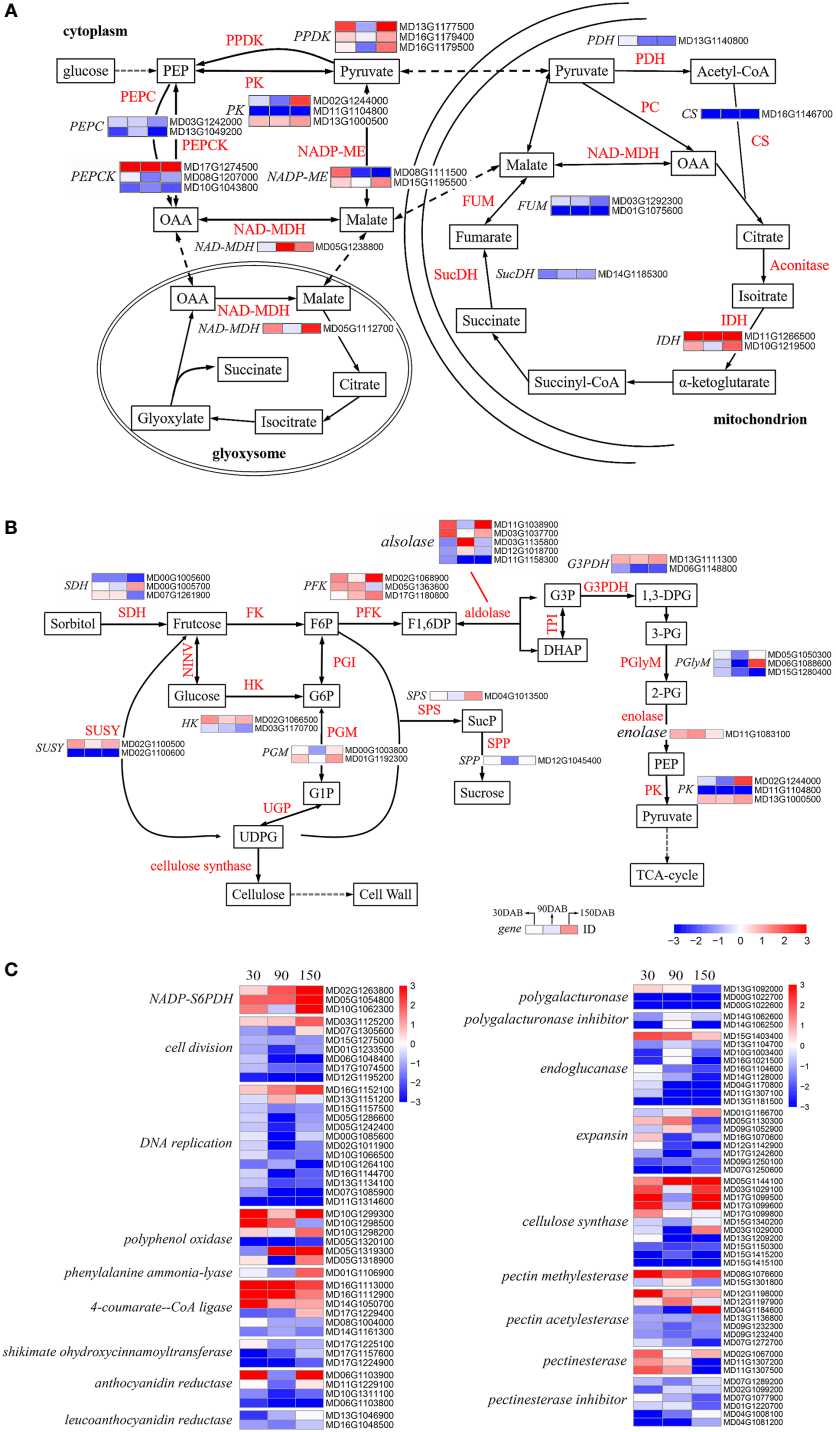
Analysis of the differentially expressed genes associated with metabolic pathways in ‘HanFu’ and ‘Huahong’. **(A)** The metabolic pathways of glucose for major acids (malic acid, fumaric acid, citric acid, succinic acid, oxalic acid) in apple fruit. **(B)** The metabolic pathways of glucose, fructose, sorbose, and sucrose in apple fruit. **(C)** A heat map of differentially expressed genes (DEGs) involved in cell wall synthesis and genes encoding other genetic regulatory pathways. A heatmap showing log2-Fold changes of DEGs (‘HanFu’ vs. ‘Huahong’).

In the glycolytic pathway, glucose is progressively cleaved to phosphoenolpyruvate (PEP), which is catalyzed by pyruvate kinase (PK, EC 2.7.1.40) (Wulfert et al., 2020; Zhang et al., 2020). We identified three genes that were differentially expressed between the two cultivars, namely, MD13G1000500, MD02G1244000 and MD11G1104800 (**Table S12** and **Figure 7A**). Pyruvate phosphate double kinase (PPDK, EC 2.7.9.1) is the rate-limiting enzyme of the C4 pathway in plants, in which pyruvate is catalyzed to produce PEP (Wang et al., 2008; Shi et al., 2020). Interestingly, all of the three DEGs encoding PPDK (MD16G1179400, MD13G1177500 and MD16G1179500) were observed to show a higher level of expression in HF at 30 and 150 DAB, thereby tending to indicate the heightened activity of these genes during the early and late stages of apple fruit development (**Table S12** and **Figure 7A**). From the cell cytoplasm, pyruvate crosses the mitochondrial membrane and enters the mitochondrial matrix, wherein it is further catalyzed in the tricarboxylic acid (TCA) cycle. Among the DEGs, we identified seven key genes associated with the TCA cycle, including pyruvate dehydrogenase (PDH, EC 1.2.4.1), citrate synthase (CS, EC 2.3.3.1), isocitrate dehydrogenase (IDH, EC 1.1.1.42), succinate dehydrogenase (SucDH, EC 1.3.5.1), and fumarate hydrolase (FUM, EC 4.2.1.2) (**Table S12** and **Figure 7A**). Interestingly, with the exception of IDH, all these DEGs exhibited higher expression in HH during all stages of development (**Table S12** and **Figure 7A**). Of the two IDHs, the gene expression (FPKM) of MD11G1266500 approached 5.94 at 30 DAB and the expression of both was higher in HF at all developmental stages (**Table S12**). Oxaloacetic acid (OAA) is catalyzed by NAD-malate dehydrogenase (NAD-MDH, EC 1.1.1.37) to malate, which passes through the mitochondrial membrane into the cytoplasm. Simultaneously, pyruvate is converted to malate by the action of the NADP-malate enzyme (NADP-ME, EC 1.1. 1.40). Malate generated by both pathways is re-oxidized to OAA by NAD-MDH and to PEP by the action of phosphoenolpyruvate carboxykinase (PEPCK, EC 4.1.1.31) (Malone et al., 2007; Shen et al., 2017; Famiani et al., 2018), completing the first step of gluconeogenesis (**Figure 7A**). We observed notable differences in the expression pattern of the DEGs associated with malate metabolism in mitochondria, with almost all these DEGs exhibiting higher expression in HF (**Table S12**). All our observations indicated that there are systematical differences regarding the biosynthesis of acids.

With respect to the biosynthesis of sugars, we identified a total of 28 DEGs in the investigated pathways (**Table S12** and **Figure 7B**), although failed to detect any consistent difference between the two cultivars, thereby tending to indicate a more complex underlying genetic basis for the biosynthesis of sugars. In addition, we found 96 DEGs with differential expression in the analysis of metabolic pathways (**Figure 7C**). These DEGs are associated with various metabolic pathways such as cell wall synthesis, polyphenol synthesis, etc.

### QTL mapping analysis and candidate gene prediction

On the basis of QTL mapping analysis, we identified 7 significant QTLs distributed among four chromosomes that were associated with sugars, with phenotypic variation explained (PVE) values of between 13.3% and 25.7%. Among these QTLs, four were found to explain more than 10% of the phenotypic variation (Fru06.1, Fru09.1, Suc04.1, and Glu09.1), and the remaining three explained more than 20% (Fru06.2, Glu06.1, Sor08.1) (**Table 1**). The physical location of these QTL regions in the genome was determined based on markers within the QTL regions. The number of genes within these QTLs ranged from 45 to 568, totaling 2,362 candidate genes for soluble sugars (**Table S14**).

**Table 1:** Summary statistics for the classification of significant QTL for sugars and acids.

Trait	QTLs	Chromosome	Start	End	Size_of_QTL	LOD	PVE	Number of genes
malate	Ma01.1	Chr01	0.32	6.79	6.47M	2.61	13.4	76
malate	Ma01.2	Chr01	2.05	2.05	0.00M	2.56	13.1	0
malate	Ma01.3	Chr01	5.64	5.64	0.00M	2.56	13.1	0
malate	Ma05.1	Chr05	30.77	32.01	1.24M	2.98	18.5	107
malate	Ma05.2	Chr05	31.39	32.96	1.57M	2.52	12.9	148
malate	Ma05.3	Chr05	32.39	34.37	1.98M	2.52	12.9	139
malate	Ma08.1	Chr08	9.42	19.51	10.09M	2.94	14.9	476
malate	Ma08.2	Chr08	12.57	19.51	6.94M	3.03	16.3	261
malate	Ma08.3	Chr08	13.49	18.54	5.05M	2.95	15.7	161
malate	Ma08.4	Chr08	16.05	25.85	9.80M	2.8	14.2	372
malate	Ma08.5	Chr08	19.47	20.80	1.33M	2.72	14.3	46
malate	Ma08.6	Chr08	21.33	23.31	1.97M	2.54	13.4	86
fumarate	Fu08.1	Chr08	3.78	3.96	0.18M	3.11	23	15
fumarate	Fu08.2	Chr08	16.37	22.04	5.67M	3.9	27.3	191
fumarate	Fu08.3	Chr08	20.30	24.59	4.29M	3.51	17.8	180
fumarate	Fu09.1	Chr09	10.66	10.66	0.00M	21.46	86	0
citrate	Ci14.1	Chr14	1.75	10.24	8.49M	3.47	18	568
citrate	Ci17.1	Chr17	0.37	1.25	0.88M	3.96	19.5	89
succinate	Su05.1	Chr05	31.39	35.72	4.33M	4.38	21.3	345
succinate	Su05.2	Chr05	33.97	38.02	4.05M	4.04	19.9	361
succinate	Su14.1	Chr14	9.97	9.97	0.00M	3.51	48.4	0
succinate	Su14.2	Chr14	11.20	16.44	5.24M	4.15	50.6	131
oxalate	Ox06.1	Chr06	36.81	37.10	0.30M	2.72	22.2	36
oxalate	Ox09.1	Chr09	12.35	14.49	2.14M	2.5	13.3	146
oxalate	Ox14.1	Chr14	18.81	18.81	0.00M	2.72	14.6	0
fructose	Fru06.1	Chr06	8.39	26.86	18.47M	3.09	18.5	568
fructose	Fru06.2	Chr06	32.97	36.59	3.62M	3.49	25.7	356
fructose	Fru09.1	Chr09	34.47	35.36	0.89M	3.27	16.8	45
sucrose	Suc04.1	Chr04	0.05	4.73	4.67M	3.61	18.2	368
glucose	Glu06.1	Chr06	8.39	26.86	18.47M	2.93	22.4	568
glucose	Glu09.1	Chr09	19.93	30.01	10.08M	2.54	13.3	218
sorbitol	Sor08.1	Chr08	0.09	2.04	1.95M	3.99	22.1	239

Comparatively, we identified a total of 25 significant QTLs associated with the five assessed acids distributed across seven chromosomes, with (PVE) values ranging from 12.9% to 86%. Among these, 18 main-effect QTL explained more than 10% of the phenotypic variation and 7 explained more than 20% (Fu08.1, Fu08.2, Fu09.1, Su05.1, Su14.1, Su14.2, and Ox06.1, Ox14.1) (**Table 1**). Interestingly, the QTL Su14.2 mapped to chromosome Chr14 (**Table S13**), corresponding to 11.20–16.44 Mb on the genome, exhibited the highest LOD score (4.15) and PVE value (50.6%) (**Table 1**), thereby indicating that this region of Chr14 has a strong genetic effect regarding this phenotype.

Using RNA-seq analysis, we predicted the candidate genes within the detected QTL regions. As indicated previously, we detected seven QTLs associated with sugar contents (sucrose, fructose, glucose, and sorbose), which collectively harbored a total of 2,363 genes. Among these, RNA-seq analysis revealed 424 DEGs within the QTL intervals (**Table S16**). As candidate genes associated with sugar content, we selected six of these DEGs based on the gene annotation information (**Table S17**). Among these, *MdBAM3* (MD06G1112400), encoding a β-amylase, is involved in starch degradation and maltose metabolism in chloroplasts (Fulton et al., 2008). *MdBAM2* (MD09G1275700) similarly encodes a β- amylase, although this appears to be less active than the *MdBAM-*encoded enzyme, and has a weaker interaction with starch and maltose (Li et al., 2009b; Monroe, 2020). *MdSPSA3*(MD04G1013500), encoding a sucrose phosphate synthase, plays an important role in sucrose synthesis as a rate-limiting enzyme that catalyzes the synthesis of sucrose from glucose and F6P (Qazi et al., 2012). *MdGUX8*(MD06G1058200) encodes a putative glucuronosyltransferase, whereas *MdPLST3*(MD09G1209000) encodes a plastid glucose transporter protein involved in the transport of glucose to the cytoplasmic matrix, and *MdAGPL1*(MD08G1027900), encoding an ADP-glucose pyrophosphorylase, plays a role in starch synthesis.

With respect to the five assessed acids (malate, fumarate, oxalate, succinate, and citrate), we detected 25 associated QTLs, harboring a total of 3,934 genes (**Table S15**), among which, 643 DEGs were identified within the QTL intervals based on RNA-seq (**Table S16**). With reference to the annotation information obtained for these DEGs, we selected three as acid-associated candidate genes (**Table S17**). Among these, *MdNADPME* (MD08G1111500) encodes an NADP-dependent malic enzyme involved in catalyzing the oxidative decarboxylation of malic acid (Shi et al., 2015; Chen et al., 2019); *MdMDHC* (MD05G1238800) encodes a malate dehydrogenase involved in the conversion of oxaloacetate to malic acid (Etienne et al., 2013); and *MdALMT9*(MD06G1214800) encodes an aluminum-activated malate transporter protein involved in the efflux of malic acid (Gao et al., 2018).

## Discussion

### The use of co-expression modules to identify candidate genes associated with sugar and acid contents in apple fruits

In this study, we performed RNA-seq analysis to identify genes showing differential expression between the two apple cultivars ‘HanFu’ and ‘Huahong’ at different stages of fruit development and used these DEGs as a basis for subsequent co-expression module analysis. By screening the modules with significant associations with the traits of interest, we accordingly identified hub genes that are speculated to play key roles in the regulation of these traits.

Among the modules characterized, the “MEpurple” module was found to be significantly associated with fruit length, weight, and sugar contents. In the model plant *Arabidopsis thaliana*, *AtMIF2* binds specifically to *AtKUN* to form a transcriptional repressor complex that inhibits the expression of *AtWUS*. Repression of this gene affects carpel number and ultimately fruit size (Bollier et al., 2018). In apple, *MdACCH1* catalyzes the terminal reaction of the ethylene biosynthesis pathway, in which ACC is converted to ethylene (Shi and Zhang, 2012), with expression reaching peak levels during the period of fruit ripening, which is consistent with our RNA-seq results. ACC can also promote plant fruit ripening by influencing the activity of plant hormones, such as salicylic acid and the growth hormone indole acetic acid (IAA) (Song et al., 2005). The “MEbrown” module obtained in this study was established to be specifically associated with fruit browning. In this regard, *MdANRPN* has been demonstrated to yield precursors required for the synthesis of proanthocyanidins or condensed tannins (Bogs et al., 2005), and also catalyzes NADPH-dependent double reduction of anthocyanins (Gargouri et al., 2009; Gargouri et al., 2010). *MdPILS5*, which encodes a novel growth hormone transporter protein, regulates intracellular growth hormone distribution (Barbez et al., 2012; Liu et al., 2018), whereas *MdCEL1* is a key enzyme involved in cellulose formation in the cell wall, which is closely associated with plant growth, xylem development, and cell wall thickening (Palomer et al., 2006; Shani et al., 2006). *MdPMEI3* encodes a pectin esterase inhibitor that regulates the demethylation of pectin in apical meristematic tissues, thereby influencing protoplast formation and foliar structure patterns (Lionetti et al., 2007). Overexpression of *AtPMEI3* has been shown to promote HG hypermethylation and influences the formation of floral primordia (Li et al., 2021). The hub gene *MdFTSHC*, detected in the “MEblue” module, is an ATP-dependent zinc metalloprotease containing an AAA (an ATPase associated with various cellular activities) and a Zn^2+^ metalloprotease structural domain that plays a key role in the hydrolysis of membrane proteins (Adam et al., 2005; Wagner et al., 2012). In the “MEred” module, the hub gene *MdXTHs* encodes a cell wall enzyme involved in the linking of xylans to oligosaccharides or other available xylan chains, and is believed to play important roles in regulating growth and development (Yokoyama et al., 2004; Becnel et al., 2006; Maris et al., 2011). *MdMES17* encodes a methyl esterase that efficiently and specifically hydrolyzes methylindole-3-acetic acid (MeIAA) to IAA (Yang et al., 2008). The roots of *Arabidopsis* plants overexpressing AtMES17 have been found to be characterized by an enhanced sensitivity to MeIAA, although not to IAA. Among genes in the “MEyellow” module, *MdABC1K8* encodes a BC1 complex kinase, and mutants of *MdABC1K8* are characterized by the production of higher levels of lipoproteins that, in conjunction with the activity of *MdABC1K7* (Manara et al., 2015), influences the synthesis or accumulation of chloroplast lipids and regulates the composition of chloroplast membranes in response to stress. The hub genes identified in the present study, based on co-expression network analysis, will provide a reference for studying the gene regulatory networks associated with fruit quality traits.

### A combination of RNA-seq analysis and QTL mapping was used to identify candidate genes associated with sugar and acid accumulation

On the basis of the co-localization of DEGs and QTL regions, we identified six sugar-associated candidate genes from among 283 DEGs with annotation information. Among these genes, *MdSPS3* encodes a sucrose phosphate synthase, the orthologs of which have been extensively studied in prunes, peaches, grapes, and tomatoes, in which it plays a catalytic role in sucrose synthesis, thereby enhance fruit sweetness. The proteins encoded by *MdBAM2* and *MdBAM3* have measurable β-amylase activity, with higher activity being detected in the latter (Fulton et al., 2008). In *Arabidopsis*, *AtBAM3* plays an important role in nocturnal starch degradation, and in *AtBAM3* mutants in which total β-amylase activity is reduced, there is a corresponding increase starch content. The β-amylase encoded by *AtBAM2* has been observed to have very low activity and a poor glucan binding capacity. Notably, in *AtBAM2*, there is no reduction in total β-amylase activity, and thus it is assumed that *AtBAM2* has no effect on amylolysis (Li et al., 2009b). In contrast to most studies, Monroe et al. (2017) found that the *AtBAM2-*encoded β-amylase had significant catalytic activity under specific physiological circumstances, a unique result that provides evidence to indicate a novel pathway for the study of *AtBAM2*. ADP-glucose pyrophosphorylase, an enzyme comprising four large and two small subunits, plays a regulatory role as the rate-limiting enzyme of the amylogenic pathway. Among these subunits, one of the large subunits (*AtAGPL3*) is capable of promoting starch synthesis (Hwang et al., 2006). Within the intervals of the 20 acid-associated QTLs, we detected 643 DEGs, three of which were identified as candidates. Among these, *MdNADPME* encodes an NADP-dependent malic enzyme involved in catalyzing the oxidative decarboxylation of malic acid (Chen et al., 2019), and has been identified as one of the essential enzymes for malic acid metabolism. It is accordingly speculated that differences in the accumulation of malic acid in ripe apples could be attributable to the differential expression of this gene (Shi et al., 2015). Of the other two candidates, the malate dehydrogenase encoded by *MdMDH* is a key enzyme in malate metabolism involved in the conversion of oxaloacetate to malate (Etienne et al., 2013), whereas *MdALMT9* encodes an aluminum-activated malate transporter protein involved in the efflux of malate and citrate, and plays an important role in the aluminum tolerance of plants (Liu et al., 2009; Kochian et al., 2015; Gao et al., 2018).

Although the candidate genes identified based on a combined QTL mapping and RNA-seq approach differ from the hub genes determined using WGCNA, we nevertheless believe these candidate genes to be informative from the perspective of characterizing changes in sugar and acid contents during the different stages of fruit development.

## Data availability statement

The data presented in the study are deposited in the China National Center for Bioinformation (https://www.cncb.ac.cn/) repository, accession number PRJCA012452.

## Author contributions

FS, JZ and XW designed the study and methodology. XW, CH, SL, CX, HZ, XH, RW performed the analyses and discussed the results. JZ and CH wrote the manuscript draft. FS performed writing-review, editing and supervision. All authors contributed to the article and approved the submitted version.

## Funding

This work was supported by the Beijing Natural Science Foundation (6214037), the Shanxi Province Natural Science Basic Research Program General Project (Surface) (Grant No. 2022JM-116), the National Natural Science Foundation of China (Grant No. 32260742), the Project of the Shaanxi Provincial Key Laboratory of Jujube (Grant No. sxhzdsys-zj21-03), and the Project of Yan’an Science and Technology (Grant No. SL2019ZCNY-002).

## Conflict of interest

The authors declare that the research presented in this paper was conducted in the absence of any commercial or financial relationships that could be construed as a potential conflict of interests.

## Publisher’s note

All claims expressed in this article are solely those of the authors and do not necessarily represent those of their affiliated organizations, or those of the publisher, the editors and the reviewers. Any product that may be evaluated in this article, or claim that may be made by its manufacturer, is not guaranteed or endorsed by the publisher.
