# Supplementary material for: Characterization of the genetic and regulatory networks associated with sugar and acid metabolism in apples via an integrated strategy
Source: Front Plant Sci. 2022 Nov 17;13:1066592. doi: 10.3389/fpls.2022.1066592 (PMC9712955; doi:10.3389/fpls.2022.1066592)
Supplement: Supplementary file 1 [file DataSheet_1.docx]

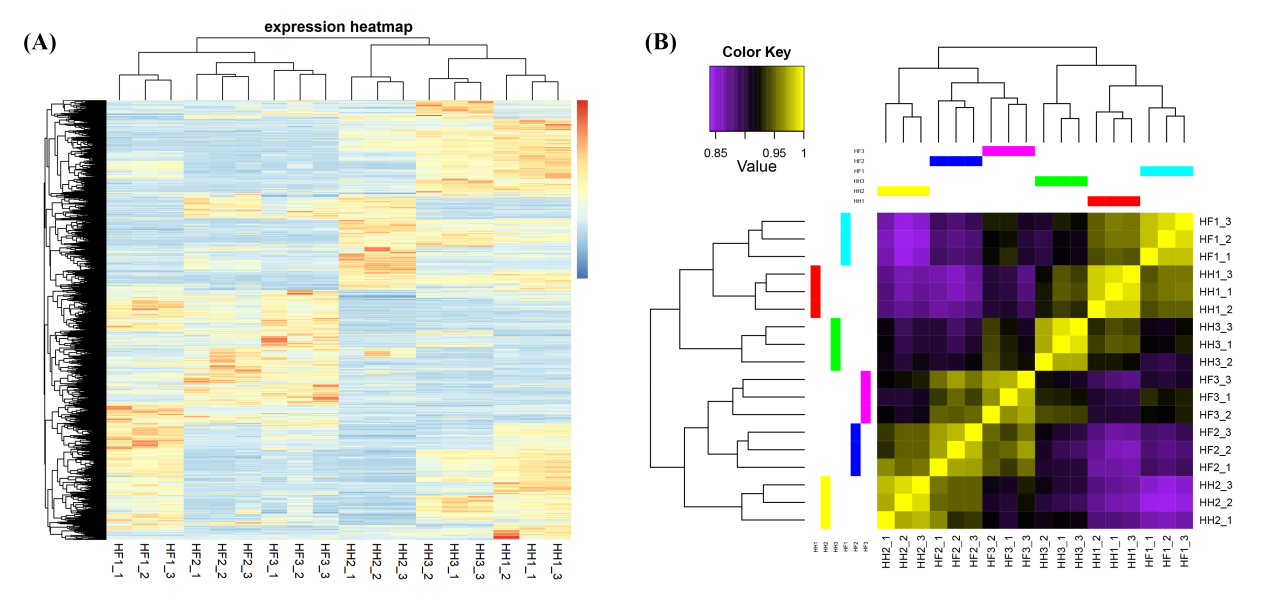


**Figure S1. Correlation between the replicates of different stages of fruit development in the two cultivars.** (A) Hierarchical cluster analysis showed the similarity of expression profiles among samples. (B) Pearson’s correlation coefficients (PCC) analysis of RNA-seq data from ‘HH’ and ‘HF’. Both hierarchical and PCC analysis based on the gene expression of all samples. HH1, HH2 and HH3 represent 30DAB, 90DAB and 150DAB of ‘HH’, respectively. So is ‘HF’.

**
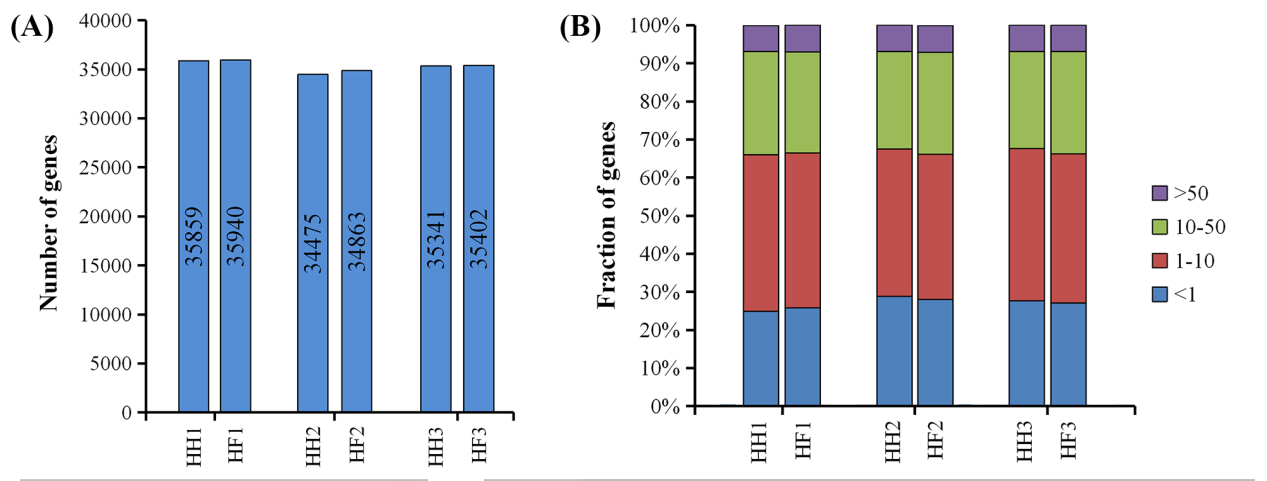
**

**Figure S2. Gene expression in ‘HH’ and ‘HF’.** Total number of genes expressed (A) and fraction of genes expressed at different expression levels (based on FPKM) (B) in different stages of development in ‘HH’ and ‘HF’ are shown in the bar graphs. HH1, HH2 and HH3 represent 30DAB, 90DAB, and 150DAB of ‘HH’, respectively. So is ‘HF’.


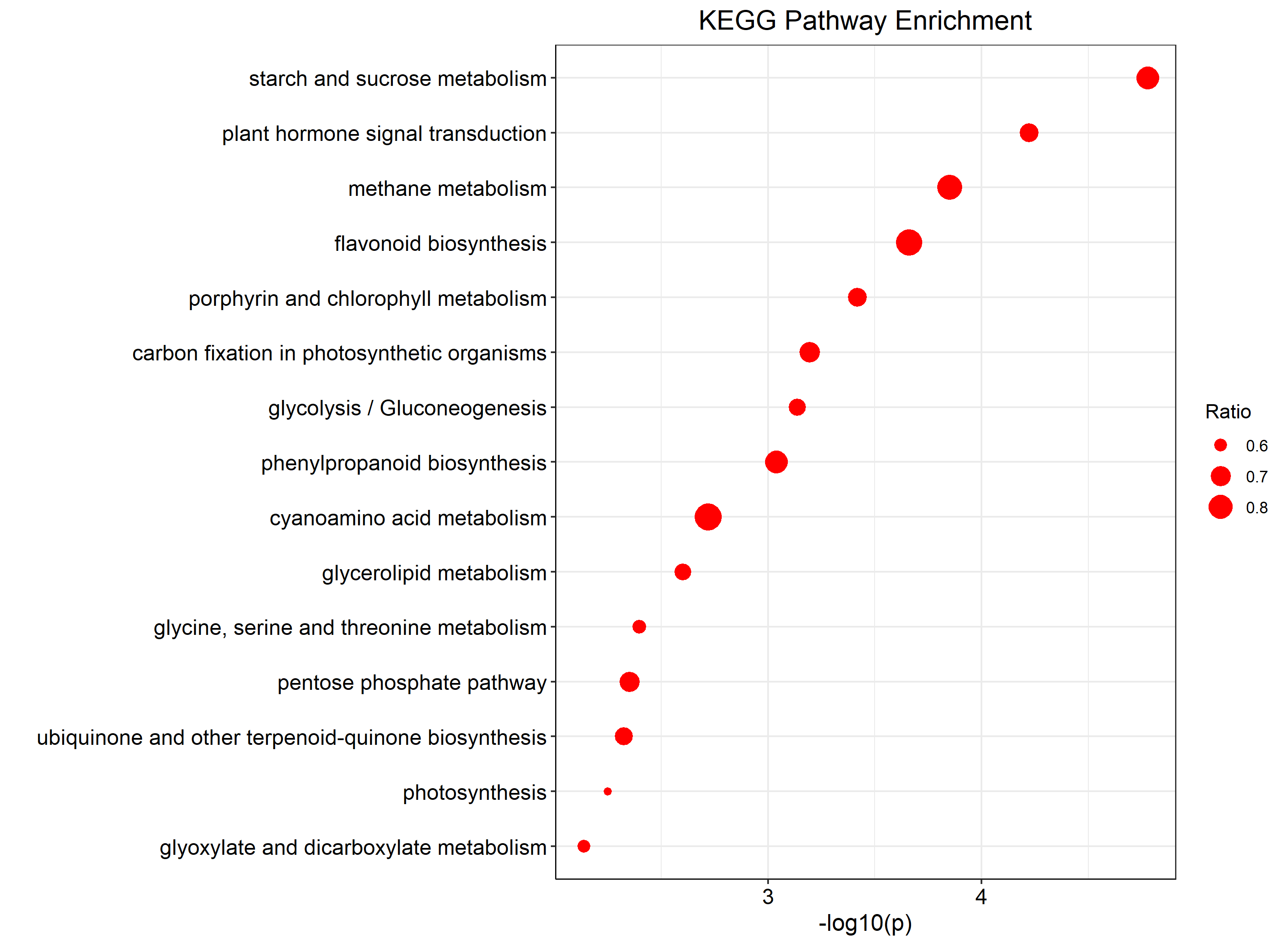


**Figure S3. KEGG pathway enrichment analysis of DEGs between ‘HH’ and ‘HF’.** The size of bubbles indicates the ratio of the number of DEGs in the pathway to that of total genes identified. The abscissa is the value of -log_10_(p).


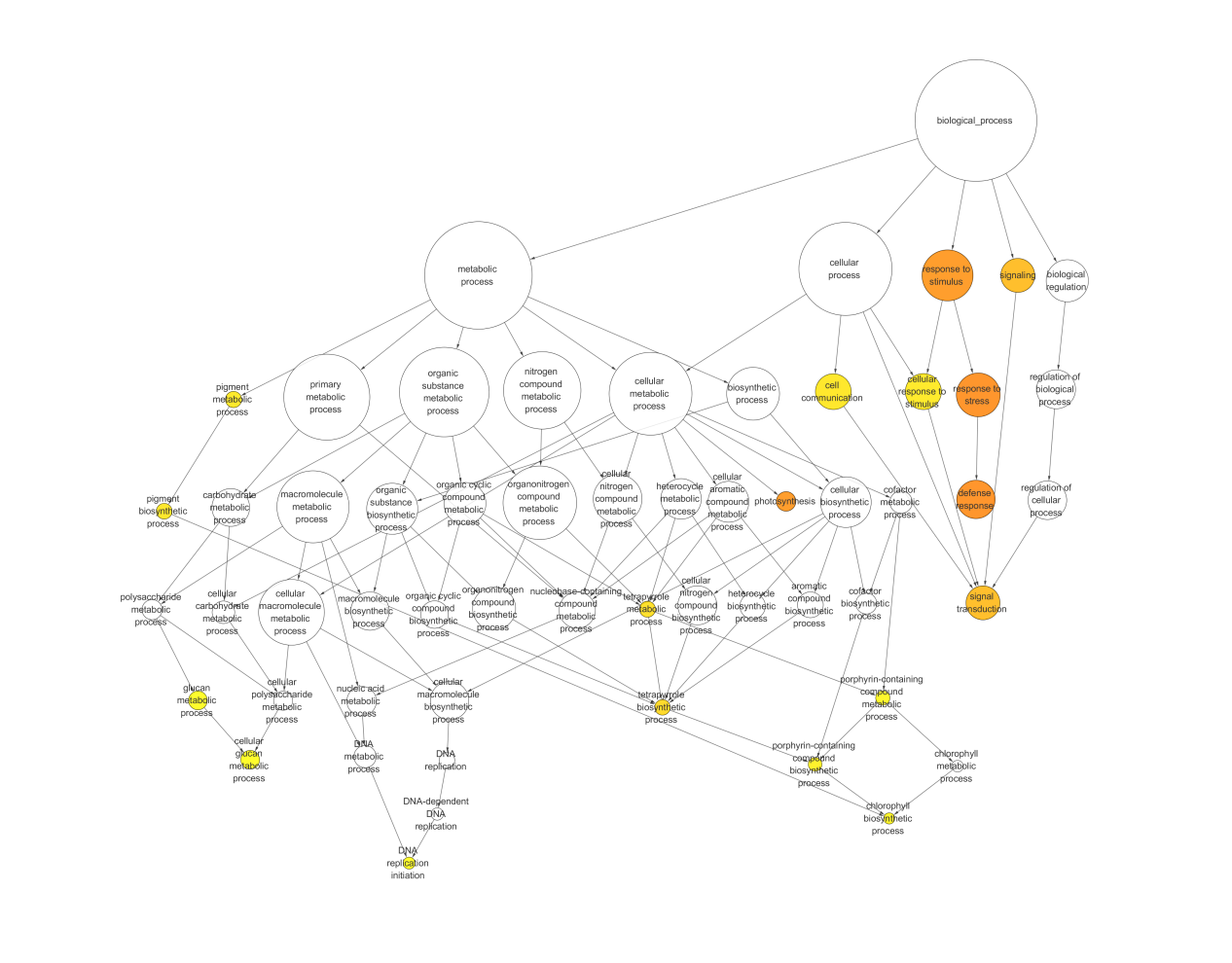


**Figure S4. GO enrichment analysis of DEGs between ‘HH’ and ‘HF’.**

**
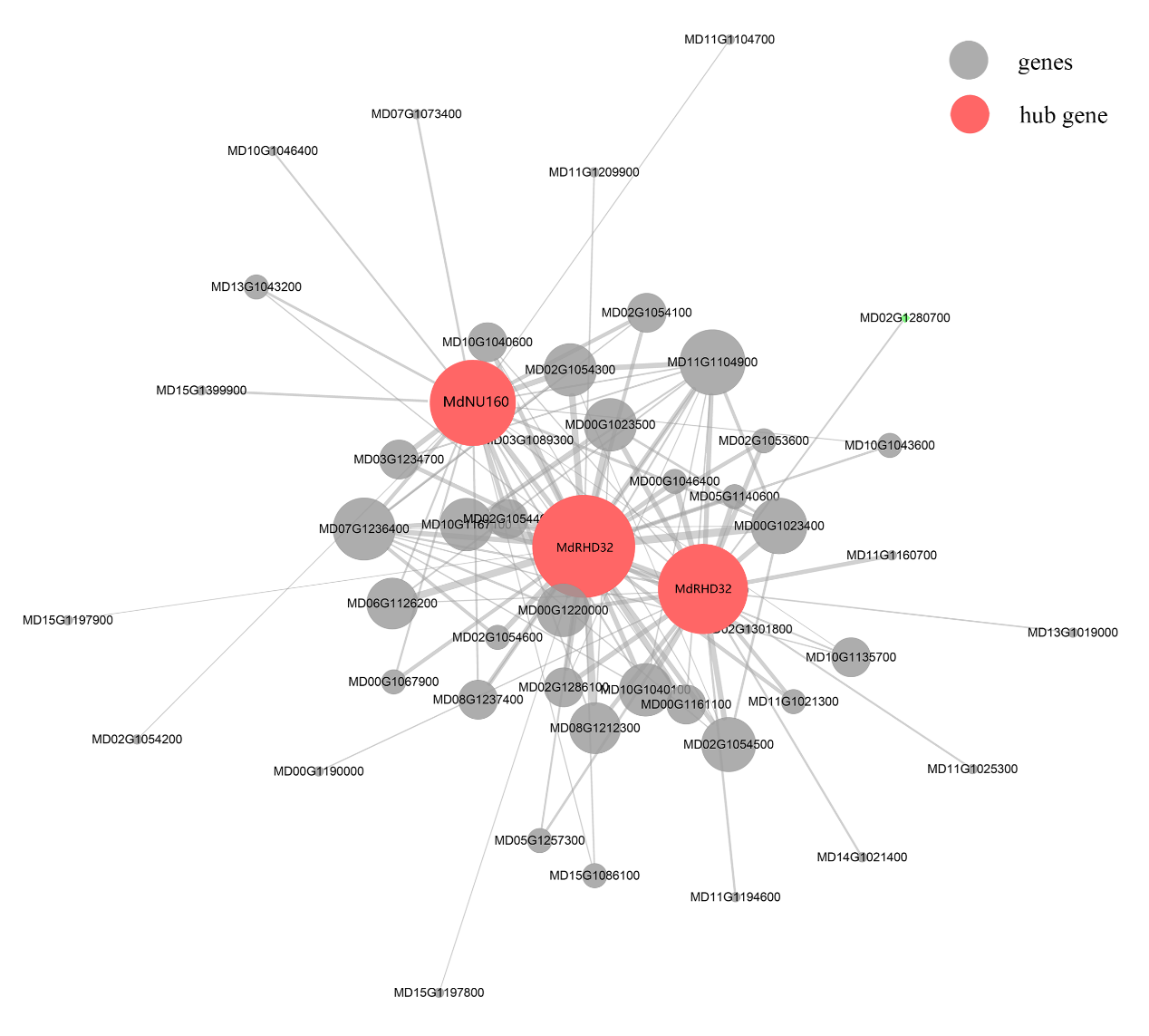
**

**Figure S5. Co-expression network of “MEblack” module.** The circulars filled by darkgrey and red indicate genes and hub genes in this module, respectively. The width of edge shows the weight value between different genes. The larger width corresponding to the larger weight.

**
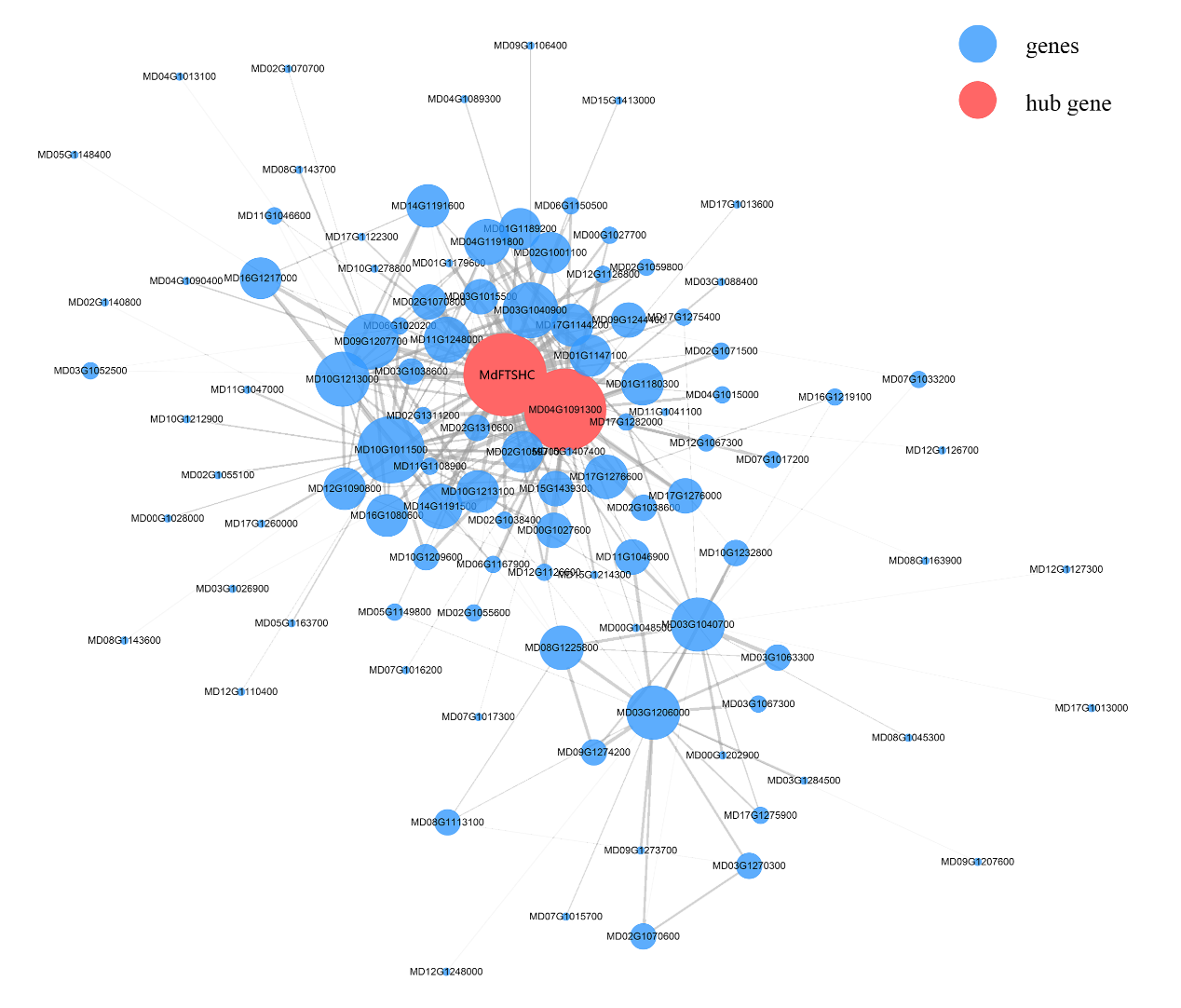
**

**Figure S6. Co-expression network of “MEblue” module.** The circulars filled by blue and red indicate genes and hub genes in this module, respectively. The width of edge shows the weight value between different genes. The larger width corresponding to the larger weight.

**
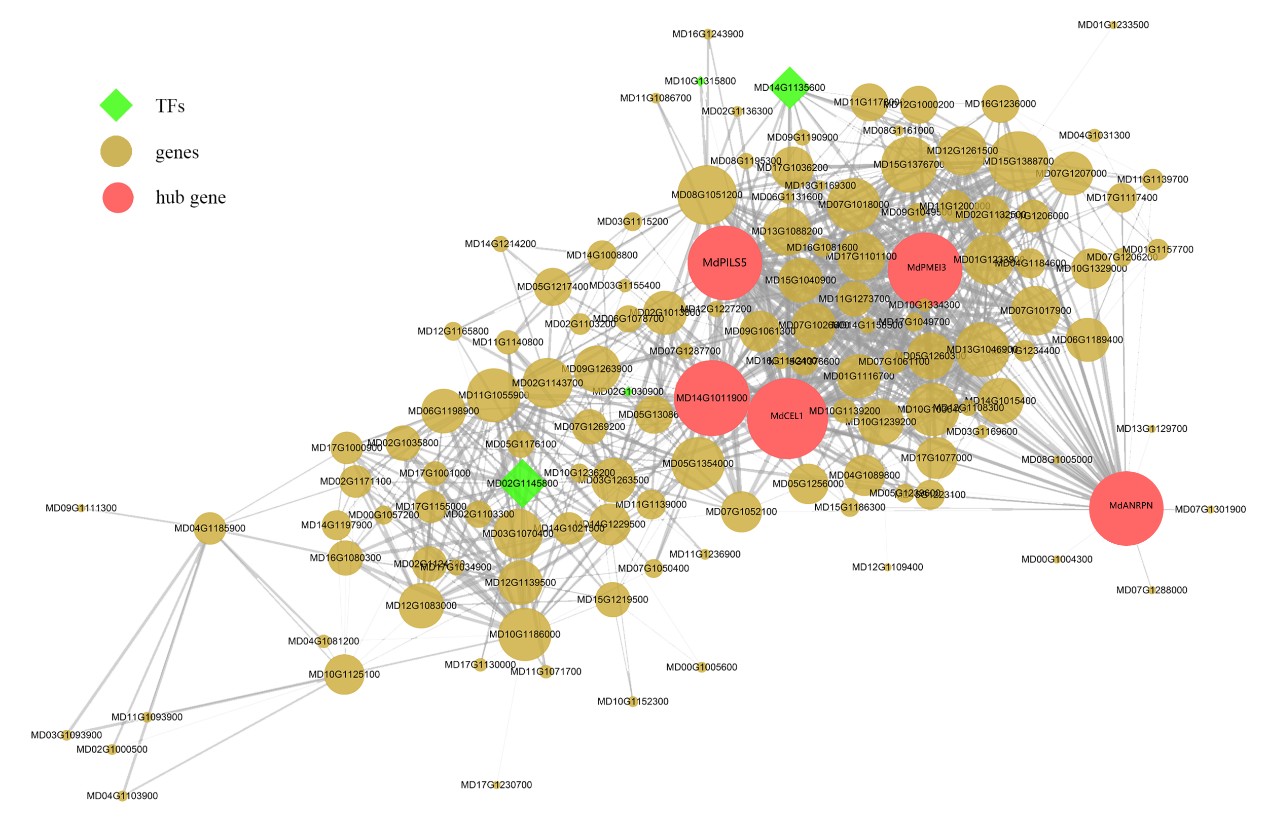
**

**Figure S7. Co-expression network of “MEbrown” module.** The green diamond indicates TFs. The circulars filled by brown and red indicate genes and hub genes in this module, respectively. The width of edge shows the weight value between different genes. The larger width corresponding to the larger weight.

**
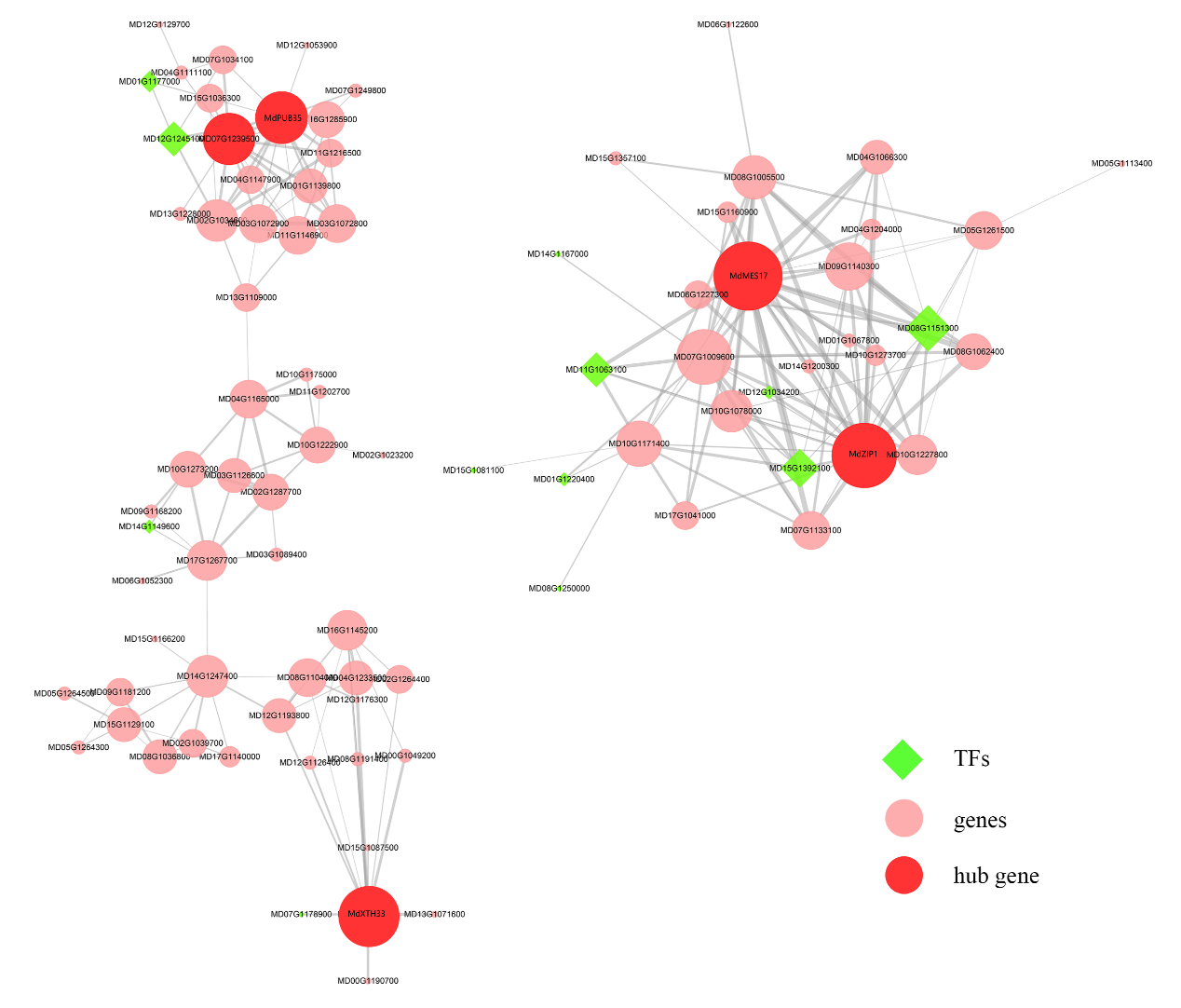
**

**Figure S8. Co-expression network of “MEred” module.** The green diamond indicates TFs. The circulars filled by lightred and darkred indicate genes and hub genes in this module, respectively. The width of edge shows the weight value between different genes. The larger width corresponding to the larger weight.

**
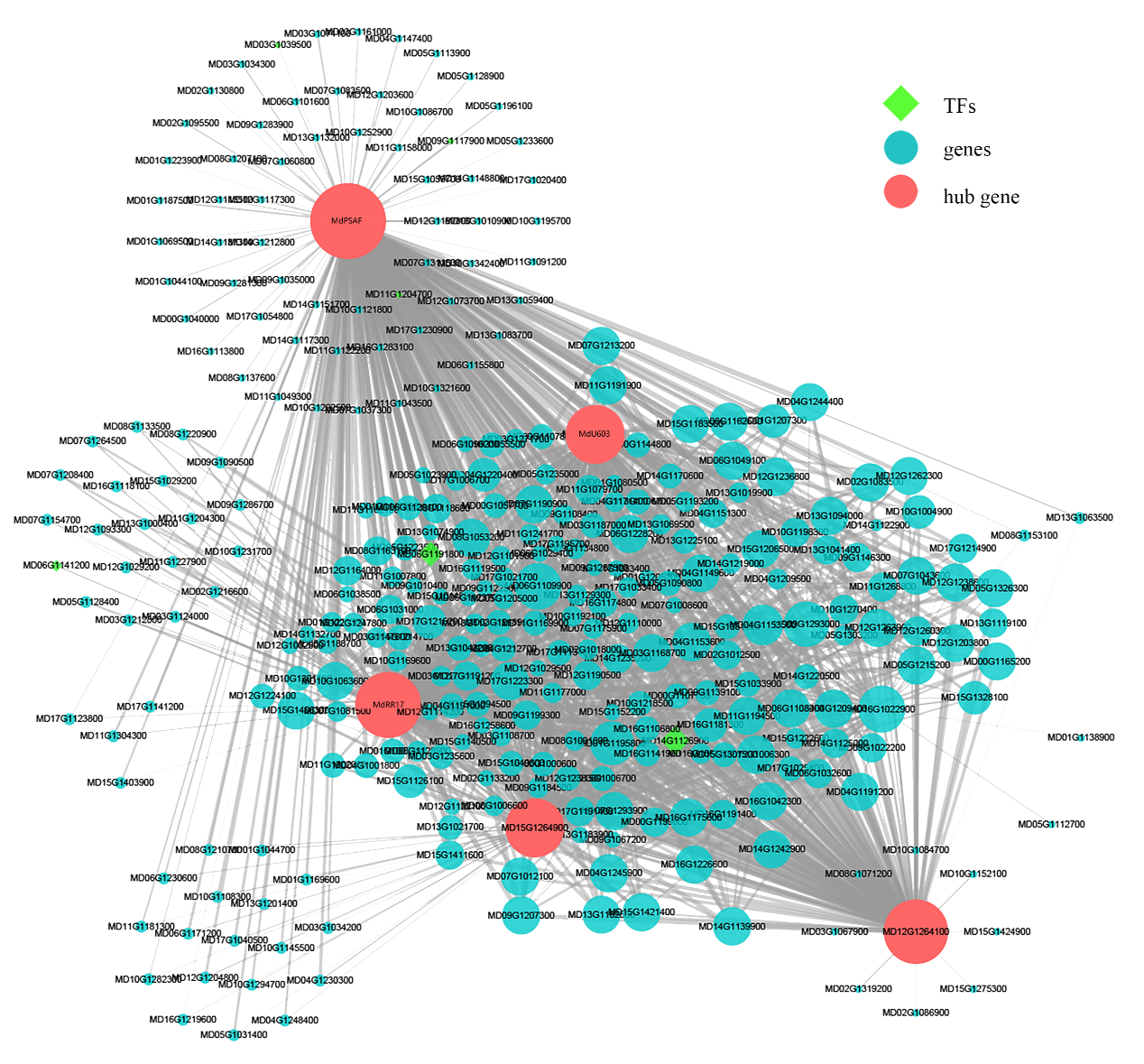
**

**Figure S9. Co-expression network of “MEturquoise” module.** The green diamond indicates TFs. The circulars filled by turquoise and red indicate genes and hub genes in this module, respectively. The width of edge shows the weight value between different genes. The larger width corresponding to the larger weight.

**
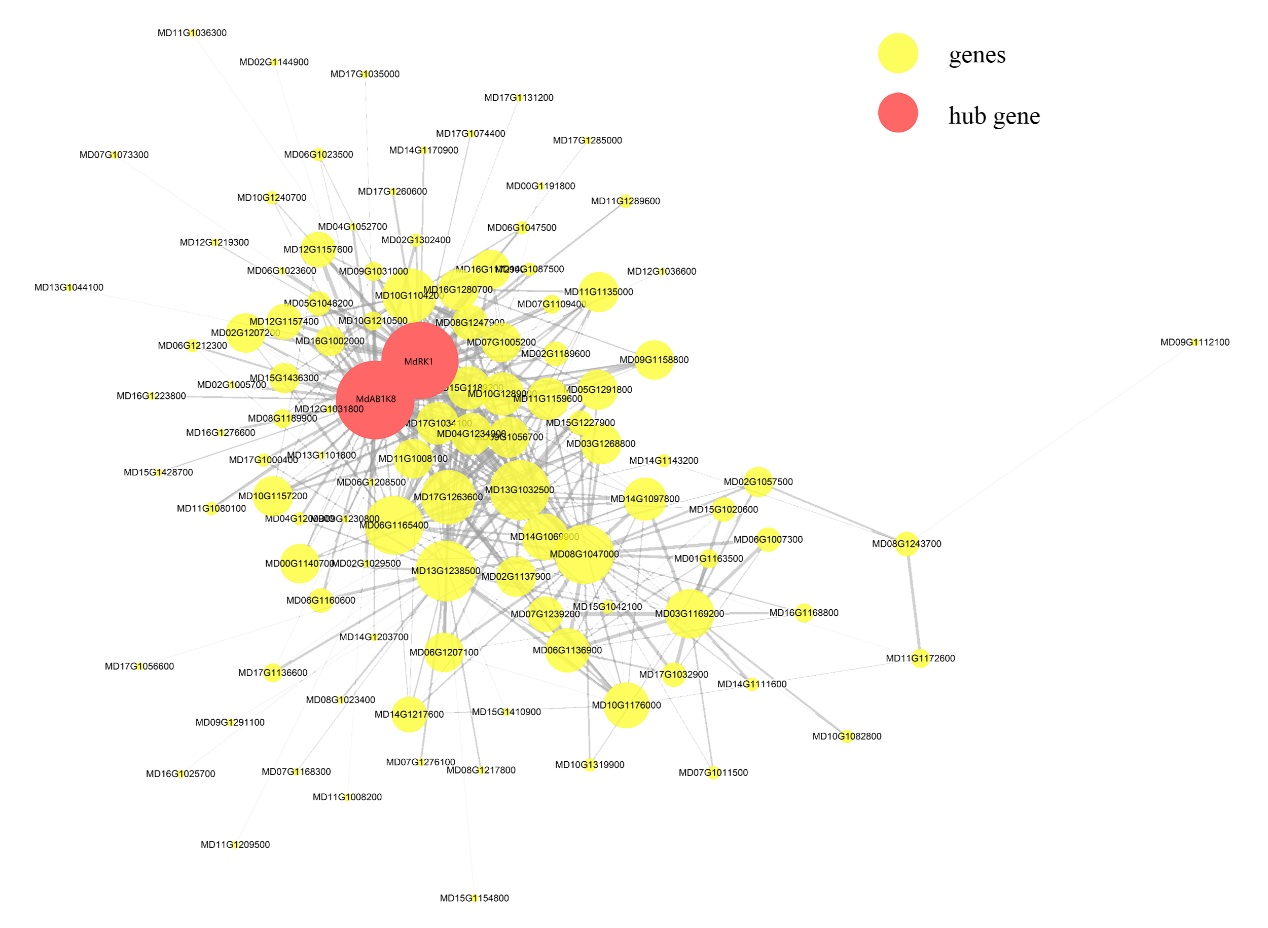
**

**Figure S10. Co-expression network of “MEyellow” module.** The circulars filled by yellow and red indicate genes and hub genes in this module, respectively. The width of edge shows the weight value between different genes. The larger width corresponding to the larger weight.
